# Sex differences in dendritic spine density and morphology in auditory and visual cortices in adolescence and adulthood

**DOI:** 10.1038/s41598-020-65942-w

**Published:** 2020-06-10

**Authors:** Emily M. Parker, Nathan L. Kindja, Claire E. J. Cheetham, Robert A. Sweet

**Affiliations:** 1 Center for Neuroscience, University of Pittsburgh, Pittsburgh, USA; 2 Translational Neuroscience Program, University of Pittsburgh, Pittsburgh, USA; 3 Department of Psychiatry, University of Pittsburgh, Pittsburgh, USA; 4 Department of Neurobiology, University of Pittsburgh, Pittsburgh, USA; 5 Center for the Neural Basis of Cognition, Pittsburgh, USA

**Keywords:** Immunohistochemistry, Spine structure

## Abstract

Dendritic spines are small protrusions on dendrites that endow neurons with the ability to receive and transform synaptic input. Dendritic spine number and morphology are altered as a consequence of synaptic plasticity and circuit refinement during adolescence. Dendritic spine density (DSD) is significantly different based on sex in subcortical brain regions associated with the generation of sex-specific behaviors. It is largely unknown if sex differences in DSD exist in auditory and visual brain regions and if there are sex-specific changes in DSD in these regions that occur during adolescent development. We analyzed dendritic spines in 4-week-old (P28) and 12-week-old (P84) male and female mice and found that DSD is lower in female mice due in part to fewer short stubby, long stubby and short mushroom spines. We found striking layer-specific patterns including a significant age by layer interaction and significantly decreased DSD in layer 4 from P28 to P84. Together these data support the possibility of developmental sex differences in DSD in visual and auditory regions and provide evidence of layer-specific refinement of DSD over adolescent brain development.

## Introduction

Dendritic spines are the predominant postsynaptic sites of excitatory input onto pyramidal cells in the cerebral cortex. Neuroscience pioneer Santiago Ramón y Cajal discovered dendritic spines in 1890^1,2^. In the 85 years between Ramón y Cajal’s death and the present day, we have learned a great deal about these micron-sized dendritic protrusions. The canonical dendritic spine is a mushroom-shaped structure protruding from the shaft of a dendrite, supported by a dynamic actin cytoskeleton, with a narrow neck and large, bulbous head. This spine contacts a single pre-synaptic axon terminal and contains the constitutive molecular machinery, receptors, channels and signaling molecules, required for transmitting incoming glutamatergic signals to the dendritic shaft. The number of dendritic spines on a neuron and the morphology of single spines are altered via actin remodeling as a consequence of synaptic plasticity and circuit refinement that occur during neurodevelopment or as a result of sensory experience. Such plasticity and structural remodeling generate substantial diversity in spine number and morphology through a myriad of context- and activity-dependent mediators^3–6^.

A growing body-of-work has established that dendritic spine density (DSD) significantly differs based on sex. Sex is an important biological variable that has recently become an important priority in biomedical research^7^. Sex differences in DSD in adult animals have so far been reported in subcortical brain regions and medial prefrontal cortex. DSD is significantly increased in female rats in the posterodorsal medial amygdala, nucleus accumbens, CA1 hippocampus (during proestrus), arcuate nucleus of the hypothalamus and medial prefrontal cortex. DSD is significantly lower in female rats in two regions of the hypothalamus, the preoptic area and the ventromedial nucleus^8–23^. DSD was recently reported to be increased on apical dendrites in medial prefrontal cortex in female mice^16^. To our knowledge, there is yet no existing published data providing evidence for sex differences in morphology or DSD in mouse auditory and visual sensory regions.

Spine formation and morphology are altered as a result of sensory experience. Sensory cortex adapts as diverse sensory stimuli shape perception and motor planning^24^. *In vivo* calcium imaging experiments reveal that visual and auditory cues evoke Ca^2+^ signaling cascades in individual dendritic spines in first-order sensory areas including primary visual and primary auditory cortices^25,26^. Ca^2+^ signaling in activated spines leads to activity-dependent actin remodeling and altered spine morphology^27^. Long-term potentiation has been shown to precipitate spine head enlargement^28–30^, whereas long-term depression precipitates spine shrinkage^29,31^. Sensory deprivation experiments demonstrate that sensory cues are required for normal patterning of dendritic spines over neurodevelopment, including reduction in dendritic spine number observed over the adolescent period^27^. A caveat of many of these studies is that they included male animals only. It remains unclear if interplays between sensory experience and alterations to spine density and morphology take place over adolescent brain development in sensory regions in female animals, as they have been shown to in males.

The goal for the current study was to characterize dendritic spines in male and female mice at the start of adolescence (P28) and in early adulthood (P84) to identify potential sex differences in spine complement and synaptic remodeling that take place during adolescence. We focus on sensory regions of the posterior cortex, first-order sensory areas: primary auditory cortex (A1), primary visual cortex (V1), plus secondary auditory cortex (A2), secondary visual cortex (V2), and temporal association cortex (TeA). Our data reveal evidence for lower DSD in auditory and visual regions of female compared to male mice for the very first time, with this effect appearing to be driven, at least in part, by fewer short stubby, long stubby and short mushroom spines in female mice. Although age did not significantly affect mean DSD in our primary statistical model, as it has been shown to in male mice, we found a significant age by layer interaction. When examining DSD from P28 to P84 in different cortical layers separately, we found that mean DSD was significantly decreased in L4, with a trend for a reduction in L5/6 from P28 to P84. There was also a trend level reduction in long mushroom spine density from P28 to P84, providing additional evidence of synaptic remodeling over the adolescent period.

## Results

### Dendritic spine density does not differ by region

Dendritic spines in five adjacent regions: A1, A2, V1, V2 and TeA were assessed in the current study, and regional identity of each pyramidal cell was estimated using anatomical landmarks and Franklin and Paxinos demarcations^32^. Region did not significantly impact DSD (F = 1.829, DF = 4, p = 0.131) after Bonferroni adjustment. There were no significant region by sex nor region by age interactions (Supplemental Fig. 1A).

### Dendritic spine density significantly differs based on cortical layer

Our survey included dendritic spines on pyramidal cells with cell bodies located in supragranular (layer 2/3 (L2/3)), granular (layer 4 (L4)) and infragranular (layer 5/6 (L5/6)) cortical layers of five adjacent auditory and visual cortical regions in a secondary statistical model with layer included as a fixed factor. Layer significantly impacted DSD after Bonferroni adjustment, and the laminar pattern of DSD: L2/3 > 4 = 5/6 was preserved across ages and sexes (L2/3 and 4 p = 0.001; L2/3 and 5/6 p < 0.001; L4 and 5/6 p = 0.100), without a significant sex by layer interaction (Supplemental Fig. 1B).

### Dendritic spine density is lower in females

**Figure 1:**
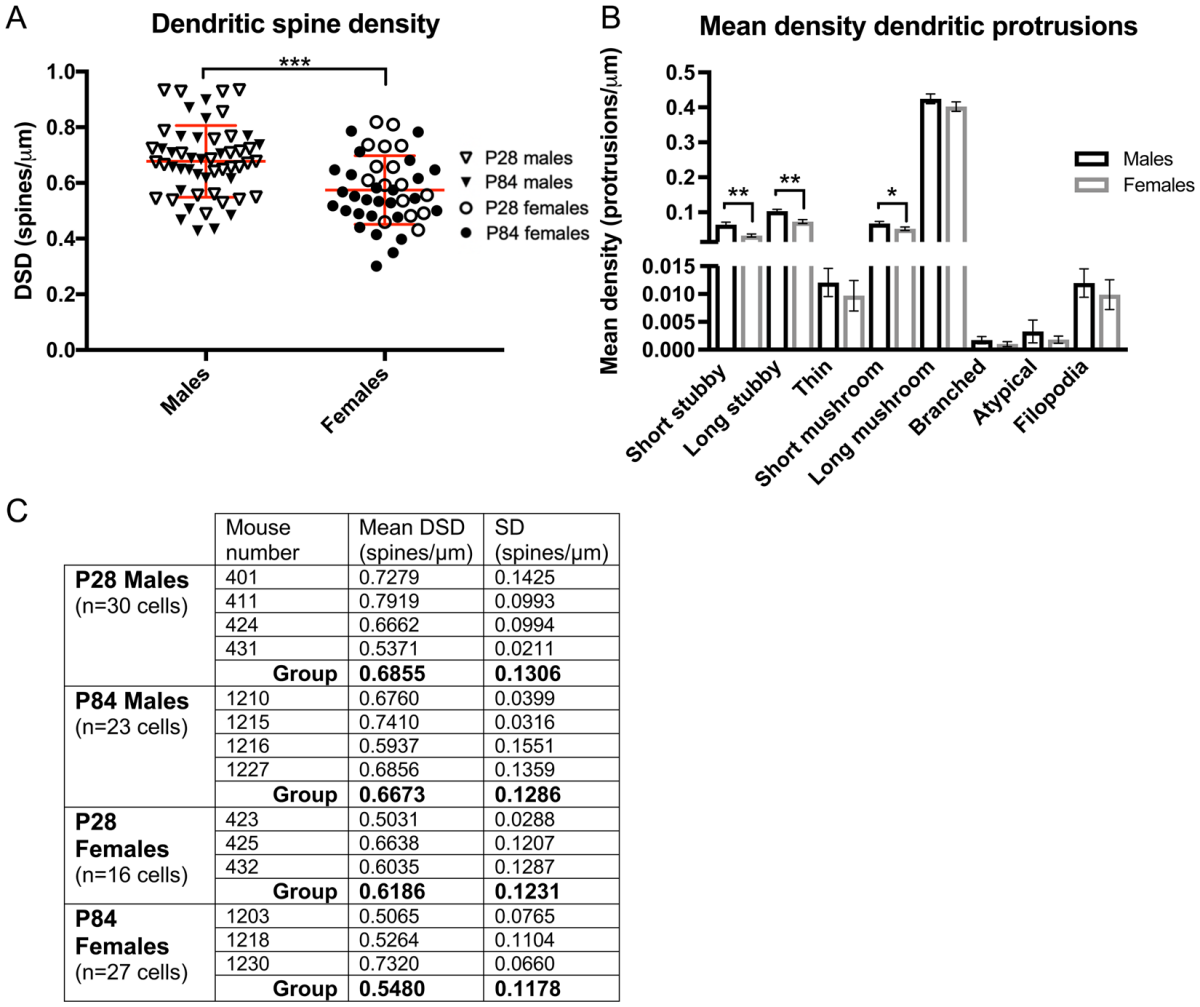
Sex differences in dendritic spine density (DSD) and mean density dendritic protrusions. (**A**) DSD is significantly reduced in female, compared to male mice (F = 14.838, DF = 1, p < 0.001). There were no significant sex by age, sex by region nor sex by layer interactions. Data points are DSD from individual neurons. Mean DSD and SD represented by red lines. (**B**) Short stubby (F = 12.408, DF = 1, p = 0.001), long stubby (F = 10.338, DF = 1, p = 0.002) and short mushroom (F = 5.834, DF = 1, p = 0.018) densities are significantly reduced in female mice with no significant age by sex interactions. Error bars = SEM. (**C**) Mean DSD for each animal. There is a trend level reduction in mean DSD in female, compared to male mice (F = 4.846, DF = 1, p = 0.055). Mean DSD is not significantly different from P28 to P84 (F = 0.005, DF = 1, p = 0.943). The age by sex interaction was not significant.

We reasoned that there would be no difference in DSD in auditory and visual brain regions in male versus female mice. In contrast to this prediction, ANCOVA (α = 0.05) revealed a highly significant decrease in DSD of neurons from female, compared to male mice (F = 14.838, DF = 1, p < 0.001) in A1, A2, V1, V2 and TeA. There were no significant sex by age, sex by region nor sex by layer interactions (Fig. 1A). In a confirmatory analysis, mean DSD was calculated for each animal (i.e. collapsing across regions and layers) and yielded complementary evidence of lower mean DSD of female mice (Fig. 1C).

Stage of estrous has been shown to modulate DSD in the ventromedial nucleus of the hypothalamus^20^ and hippocampal regions^33–35^ but not in the anterior cingulate of female rats^15^. Although we did not specifically record and evaluate DSD in female mice in different stages of the estrous cycle, we did calculate coefficient of variation (CV) to evaluate variation of DSD measurements in male versus female mice and test for homogeneity of variances using Levene’s Test^36^. If DSD significantly differed in females based on the stage of estrous cycle, one would reasonably predict that variation of DSD measured in female mice would be higher than in males. Although we did find that CV was higher in P84 females (CV = 21.49) than in males (CV = 19.29), Levene’s Test revealed that the variances were not significantly different (F(1,44)=0.010, p = 0.919).

### Stubby spine and short mushroom spine densities are lower in females

Short stubby (F = 12.408, DF = 1, p = 0.001), long stubby (F = 10.338, DF = 1, p = 0.002) and short mushroom (F = 5.834, DF = 1, p = 0.018) spine densities were significantly reduced in female compared to male mice, with no significant age by sex interactions (Fig. 1B). Short stubby, long stubby and short mushroom spines collectively make up 30.83% of the total dendritic protrusions counted in our study (Supplemental Fig. 2B). Sex did not appear to influence density of any of the other spine types, nor filopodia.

### Dendritic spine density differs across adolescence in layer-specific manner

**Figure 2:**
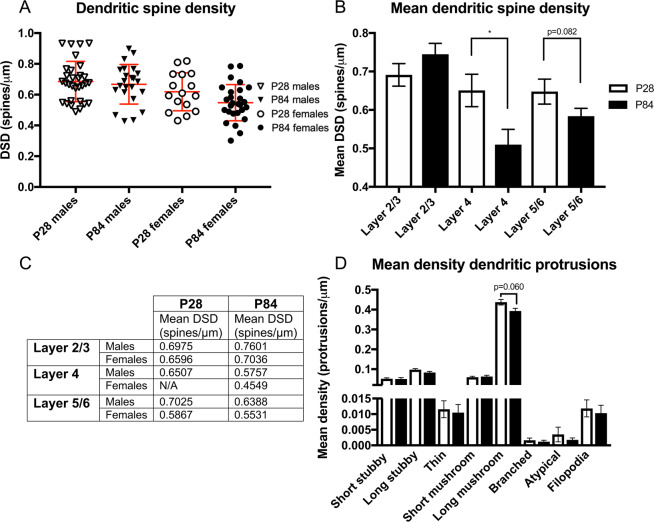
DSD and mean density dendritic protrusion findings over adolescent brain development. (**A**) DSD is not significantly changed in male and female mice from P28 to P84 (F = 0.001, DF = 1, p = 0.971). Data points are DSD from individual neurons. Mean DSD and SD represented by red lines. (**B**) There is a significant age by layer interaction (F = 0.777, DF = 2, p = 0.043). Mean DSD is unchanged from P28 from P84 in L2/3 (F = 1.516, DF = 1, p = 0.229), significantly decreased in L4 (F = 5.880, DF = 1, p = 0.026) and unchanged in L5/6 (F = 3.082, DF = 1, p = 0.086) over this period. Error bars = SEM. (**C**) Table demonstrating mean DSD of males and females at P28 and P84 show same laminar patterns of mean DSD shown in Fig. 2B; the age by sex by layer interaction was not significant. (**D**) Long mushroom mean density is nearly significantly reduced over adolescent neurodevelopment (F = 3.615, DF = 1, p = 0.060) with no significant age by sex interactions. Error bars = SEM.

We hypothesized that DSD would be significantly reduced during adolescent development, from P28 to P84, consistent with our previously published findings of reduced spine number in layers 2–4 of male mouse A1 across adolescent neurodevelopment^37^. In contrast to this hypothesis, we found no significant change in DSD over adolescent development (F = 0.001, DF = 1, p = 0.971) in auditory and visual brain regions in male and female mice in our primary statistical model (Fig. 2A). The age by sex interaction was also not significant. However, the age by layer interaction was significant (F = 0.777, DF = 2, p = 0.043). Mean DSD was significantly lower at P84 in L4 (n = 19 neurons)(F = 5.880, DF = 1, p = 0.026), with no change in mean DSD in L2/3 (n = 31 neurons)(F = 1.516, DF = 1, p = 0.229) nor in mean DSD in L5/6 (n = 47 neurons) (F = 3.082, DF = 1, p = 0.086) (Fig. 2B). The age by sex by layer interaction was not significant (Fig. 2C).

### Long mushroom spine density across adolescent development

Long mushroom spine density was lower at P84 in male and female mice. This observation was a trend that approached statistical significance (F = 3.615, DF = 1, p = 0.060) (Fig. 2D). Long mushroom spines made up 66.91% of the total dendritic protrusions counted in our study (Supplemental Fig. 2B). Densities of the other spine types and of filopodia were not significantly different comparing P28 to P84.

## Discussion

In the current study, we set out to assess DSD and dendritic spine morphology in auditory and visual brain regions of male and female mice at P28 and P84 to determine if sex differences in dendritic spines are present at these ages and to acquire a more comprehensive understanding of synaptic remodeling across adolescence. We reasoned that sex would not significantly impact DSD in the regions we surveyed given that auditory and visual cortices do not fundamentally drive sex behaviors and that there are no established links between sex hormones and spine dynamics in these regions in mice. In contrast, we show for the first time that DSD on minor basal dendritic segments of pyramidal cells in A1, A2, V1, V2 and TeA is significantly lower in female mice. Lower DSD in female mice was robust; this effect was present even after calculating mean DSD for each animal, which included DSD from neurons located in different cortical layers.

Existing published reports of lower DSD in females are limited to two brain regions: preoptic area and ventromedial nucleus of rat hypothalamus^18,20,21,23^. Preoptic area and ventromedial nucleus are hypothalamic regions that undergo sex differentiation in the juvenile period of postnatal rat development and support the emergence of sex characteristics and sexual behavior. The hormone 17 β-estradiol (E2) plays a formative role in developmental patterning in each of these areas, and this patterning leads to sex differences in DSD. In the medial preoptic area, E2 in male rats promotes prostaglandin synthesis to facilitate masculinization, which results in dendritic spine formation. In the ventromedial nucleus of male rats, E2 promotes glutamate release to facilitate defeminization, resulting in increased dendrite branching and spine formation^19^. It seems plausible that the sex differences in DSD we observed in our study occur as a consequence of sex differentiation during early developmental patterning of auditory and visual regions given that DSD was detectably lower in female mice at P28, an age at which activational effects of gonadal steroids would be limited. Whether the patterning in mouse auditory and visual brain regions is mediated by E2 through mechanisms similar to those that occur in rat hypothalamus remains to be determined.

Coefficient of variation calculations confirmed that variation in mean DSD is greater in female than in male mice, consistent the possibility that stage of estrous could mediate mean DSD in female mice. However, Levene’s Test demonstrated that the variances among males and females are not significantly different, indicating that estrous stage is unlikely to mediate mean DSD in females in our study. It should also be noted that our data do not reflect plastic changes that are associated with motherhood, including altered neuron firing properties in primary and association sensory regions;^38–45^ males and females were housed separately after weaning at P21 and none of the female mice in our study produced offspring.

Short stubby, long stubby and short mushroom spine densities were significantly reduced in female mice. Dendritic spine morphology is widely variable^3–5^. Despite this, all dendritic spines share two features: a spine head harboring synaptic machinery and a neck region connecting the spine head to the dendritic shaft. Spine head volume and spine neck diameter are thought to be regulated independently^46^. The spine neck region acts as a biochemical and electrical bottleneck. The narrower the neck region, the higher the resistance for molecules moving toward or away from the synapse. Stubby spines were defined in our study as dendritic protrusions lacking a clear distinction between the head and neck. These spines would, in theory, lack the neck resistance that other spines (like mushroom spines) can provide, and since single spines are hypothesized to play active roles in dendritic integration through linear and non-linear mechanisms^47^, lower density of stubby spines in female mice could impact integration dynamics of synaptic inputs in single pyramidal cells^48^. Future studies are required to determine if there are sex-based differences in dendritic integration or gain in cortical pyramidal cells at sites where spine morphology profiles differ in male and female mice.

We previously observed lower dendritic spine number at P84, compared to P28, in L2–4 of A1 in male mice^37^. In contrast to our previous reported findings, our current data reveal that age does not significantly impact DSD. We reason that the discrepancy in our findings could be due to one or more of the following factors: spine labeling method, sampling, and measurement. Labeling method is unlikely to account for the lack of agreement across the two studies since the co-labeling strategy employed in the previous study to identify spine objects clearly labeled GFP-positive dendritic protrusions in tissue from the current study (Supplemental Fig. 2C). In terms of sampling, we previously sampled from L2–4, counting all spine objects, whereas in the current study we sampled from L2/3, L4 and L5/6, only counting spines on proximal (minor) basal dendrites on a subset of systematically randomly sampled pyramidal cells. We observed stark differences in DSD on proximal (minor) basal dendrites of pyramidal cells located in different cortical layers (Supplemental Fig. 1B). Although spines from basilar dendrites of L5/6 neurons were included in the current but not prior study, differences in laminar sampling alone are unlikely to explain the discrepancy in the findings from the two studies since the directionality of change in mean DSD with age in L2/3 differs between the two studies. Differences in measurement seem most likely to account for the discordant results observed. In our prior study we used an immunohistochemical strategy that labelled all putative spine objects in L2–4 regardless of cell body location and location on dendritic tree. We then computed spine number which is dependent on both density of spines in tissue and tissue volume of the region of interest. Tissue volume is known to be affected by normal developmental patterning of structures other than spines themselves, including dendrite, axon, myelin and glial volumes. It seems likely that such differences in spine number/density measurement could account for the lack of agreement in results in the two studies, although it would be necessary to use both measurement methods to count spines in the same mouse subjects to confirm whether or not this is true.

Mouse strain and environmental enrichment are two additional factors that cannot be ruled out as potential contributors to discordant results. C57BL/6NJ mice were used in the prior study whereas C57BL/6 J mice were used in the current. C57BL/6 J is distinguished from C57BL/6NJ by five SNP differences and a deletion in the *Nnt* gene. Such subtle genetic differences could have accounted for the divergence in the results. Environmental enrichment (complex housing)^49^ has been shown to increase DSD in male rat occipital cortex^50–52^, supporting the possibility that environmental enrichment could have mediated DSD on basal dendrites in homologous regions in murine cortex in our study. Animals in our current study were exposed to environmental enrichment starting at P21. Interestingly, one study demonstrated that environmental enrichment increased the total length of basilar dendrites in visual cortex of male but not female rats^53^. If exposure to environmental enrichment increased DSD in addition to total dendrite length only in male^50^ but not female mice in our study, this may be able to explain why we found DSD was lower in females exposed to environmental enrichment and DSD was not significantly lower at P84 in males (compared with our prior report of reduced spine number from P28 to P84 in standard housed male mice^37^). However, although we can confirm that, unlike in our previously published study of DSD in A1, mice in the current study had access to environmental enrichment, we cannot confirm the frequency by which these mice specifically utilized this environmental enrichment nor if this enrichment had any direct impact on basal dendritic DSD.

Despite the fact that we did not find a significantly lower DSD at P84 in our current study, our data did reveal important evidence of synapse remodeling across adolescence. Given the impact of layer on DSD (Supplemental Fig. 1B), we built a secondary statistical model to further probe the impact of layer and age on DSD. This assessment revealed a significant age by layer interaction and that mean DSD is unchanged across adolescence in L2/3 (n = 31 neurons) (F = 1.516, DF = 1, p = 0.229), significantly lower at P84 in L4 (n = 19 neurons) (F = 5.880, DF = 1, p = 0.026) and lower at P84 in L5/6, although the difference was not statistically significant (n = 47 neurons) (F = 3.082, DF = 1, p = 0.086). Lower mean DSD at P84, compared to P28, in L4 is consistent with our previous finding of reduced spine number in layers 2–4 across adolescent development. Overall, these data provide evidence that different layers in auditory and visual sensory regions undergo divergent neurodevelopmental trajectories of DSD during adolescent brain development.

Region did not significantly impact DSD (Supplemental Fig. 1A), consistent with a growing body-of-work demonstrating that regional differences in DSD do not exist across adjacent or similar cortical mouse brain regions^5,46,54,55^. Despite the fact that pyramidal cells in mice and higher mammals share the same subcellular compartments and many of the same features^56^, the finding that DSD does not significantly differ across adjacent cortical regions in mice *does not* translate to non-human primates and humans, as dendrite arbor size and DSD have been shown to *differ extensively* based on regional location within cortex in these higher mammals^57–60^.

We assessed DSD on proximal basal dendrites of pyramidal cells located in different cortical layers. DSD in L2/3 was significantly higher than DSD in both L4 and L5/6, but no difference was found in DSD in L4 versus in L5/6 (Supplemental Fig. 1B). These data agree with the well-documented diversity of cortical pyramidal cell morphology, connectivity and functional properties based on the laminar location of pyramidal cell somata^56,61–65^. Heterogeneous morphology of pyramidal cells across cortical layers is thought to support the diversity of roles characteristic of dendritic spines^65^. The laminar pattern of DSD we observed was preserved across ages and sexes, supporting the notion that pyramidal cells in different layers perform specific roles in information processing within cortical circuits. One implication of these findings is a cautionary note. DSD appears to depend on the layer in which a pyramidal cell is located, consistent with previous reports^56,61–65^, and definitive evidence for synaptic remodeling across adolescence was not found until a secondary analysis was performed, which analyzed DSD in the layers separately. Future studies that assess DSD in more than one cortical layer should specifically analyze DSD in the layers separately. For studies that use immunohistochemical methods and fluorescence microscopy, a marker is needed to reliably distinguish adjacent layers from one another. This can easily be achieved by counterstaining for NeuN as others have done in previous studies^66,67^, and we have done here.

Collectively, these results provide important evidence of sex differences and layer-specific refinement of DSD over adolescent brain development in sensory brain regions located in posterior cortex. We demonstrate for the first time that DSD is lower in female mice in cortical brain regions that have not yet been discussed in the sex differences literature and are not thought to directly drive sex behavior. One may speculate that sex differences in DSD in auditory and visual cortices generate behavioral consequences. Links between divergent developmental patterning of DSD in male and female mice and behavior should be explored in future studies. Potential roles for gonadal steroids in the modification of DSD in auditory and visual brain regions in male and female mice, defined by gonadal anatomy, should also be examined.

Finally, although it is known that the organization of auditory and visual cortices are largely conserved across primates^68,69^, it remains unclear if the sex differences we observed in mouse DSD translate to higher mammals, and future studies are required to confirm this prediction. If the sex differences finding *does* translate to human, this work could inform our understanding of sex differences in normative and in abnormal adolescent neurodevelopmental trajectories. First, as discussed above, lower DSD in auditory and visual regions in females could have consequences for behavior during normative neurodevelopment. Again, this possibility must be specifically tested. Potentially more interestingly, if this finding translates to humans, this could have implications for studying the prodromal period and/or emergence of neurodevelopment disorders, for instance schizophrenia. Postmortem studies have revealed that DSD is significantly lower in adults with schizophrenia including in A1, however the relative reduction of DSD in schizophrenia did not differ by sex^70–72^. Many believe that individuals that develop schizophrenia experience accelerated spine loss during adolescent synaptic remodeling, over and beyond the normal spine reduction that occurs during this period; this accelerated reduction in DSD across adolescence may underlie, in part, the significantly lower number of spines observed in schizophrenia in adulthood^73^. Despite no apparent sex difference in DSD in schizophrenia in A1 in adult postmortem tissue, sex differences are well described in schizophrenia including mean age of onset and clinical presentation of sympoms^74,75^. The data in our current study suggest that sex differentiation in DSD occurs prior to the start of adolescence (P28) in auditory and visual brain regions in mouse, presumably prior to schizophrenia onset in humans. Further experimental work is necessary to determine if sex differentiation processes are or are not intact in auditory and visual brain regions of human individuals at-risk for developing, or those who go on to develop, schizophrenia and whether and how such differences are associated with age of onset or symptom presentation of schizophrenia.

## Methods

### Experimental animals

E16 pregnant C57BL/6 J dams were acquired from The Jackson Laboratory (Bar Harbor, ME) and singly housed in BSL-2 biocontainment in standard microisolator cages (Allentown Caging Equipment, Allentown, NJ) on a 12 h light/dark cycle with food and water provided ad libitum. The adeno-associated virus (AAV) AAV2-CaMKII-eGFP-WPRE (titer = 1.088e13gc/ml), which is designed to selectively express the fluorescent protein eGFP in glutamatergic neurons (pyramidal cells), was obtained from Penn Vector Core. AAV injectate was prepared by diluting AAV in sterile filtered 1x PBS at 1:10^68,69^. Diluted AAV was used in order to achieve sparse AAV transduction in A1, A2, V1, V2 and TeA. P0-P2 C57BL/6 J mouse pups were exposed to AAV injectate using the bulk regional AAV injection (BReVI) procedure^70^. Briefly, neonates were cryoanesthesized^71^ to induce brief hypothermia until response to toe pinch was absent. 1 μL AAV solution was injected intracranially 1 mm rostral to the left earbud and 1 mm lateral from the midline using a custom injector: a 1 mL Luer-lock syringe connected to a pulled glass micropipette with a sharp tip. Toe amputation was performed for group identification. Pups were returned to the home cage with the dam following thrombus at site of toe amputation and 10–12 m rewarming on a heating pad. Experimental mice were housed with littermates following the BReVI procedure until 3-weeks following birth (P21), at which point mice were weaned and housed with same-sex littermates until P28 or P84. Each cage of weaned animals was provided environmental enrichment (a hut and exercise wheel) at P21, in accordance with a new policy set by the Institutional Animal Care and Use Committee (IACUC) at the University of Pittsburgh. These experiments were approved by the IACUC at the University of Pittsburgh in accordance with the guidelines outlines in the USPHS Guide for Care and Use of Laboratory Animals.

### Perfusion and tissue processing

Mice were euthanized at either P28 (4 males and 3 females) or P84 (4 males and 3 females). Mice were weighed, deeply anesthetized with Nembutal (150 mg/kg) and transcardially perfused with ice-cold 1x PBS followed by 4% PFA. Brains were rapidly extracted and post-fixed in 4% PFA for 24 h and then moved to 18% sucrose for 24 h and stored at −30 °C in 30% ethylene glycol and 30% glycerol in phosphate buffer (cryoprotectant) until sectioning. 60 μm-thick coronal tissue sections were cut on a cryostat directly into 12-well plates containing cryoprotectant and then placed in −30 °C for long-term storage.

### Immunohistochemistry

**Figure 3:**
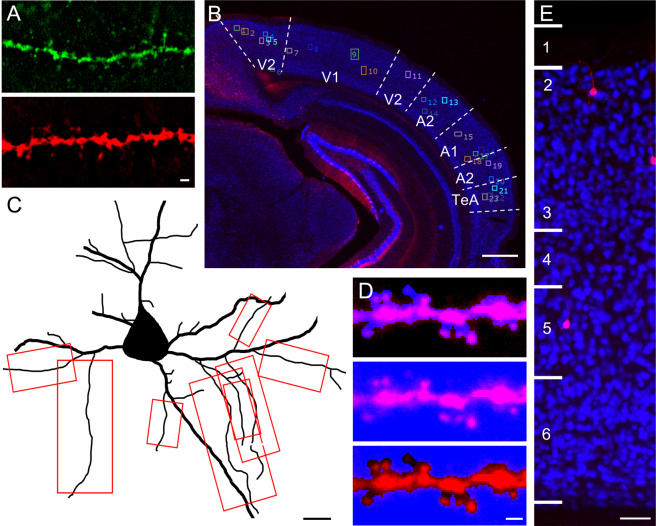
Immunohistochemical, sampling and image processing methods. (**A**) Fluorescent signal amplification using an AlexaFluor 568 secondary antibody (bottom panel) achieved superior SNR characteristics compared to amplification with AlexaFluor 488 (top panel) after no-neighbors smoothing. Scalebar = 1 μm. (**B**) Numbering strategy for fluorescent pyramidal cells in ROI of a representative 60 μm thick tissue section corresponding to plate 59 in Franklin and Paxinos’s The Mouse Brain In Stereotaxic Coordinates [49]. ROI comprises the following regions, clockwise from top left: mediomedial and mediolateral secondary visual cortex (V2), monocular and binocular primary visual cortex (V1), lateral secondary visual cortex (V2), dorsal secondary auditory cortex (A2), primary auditory cortex (A1), ventral secondary auditory cortex (A2) and temporal association cortex (TeA). Numbered neurons were imaged in random order. Scalebar = 500 μm. (**C**) Illustration of pyramidal cell with all secondary minor basal dendritic segments outlined in red meeting criteria for segment inclusion (>10 μm from soma and >=3 μm from dendrite branch point or termination). Scalebar = 10 μm. (**D**) Quantitative strategy used to exclude dim dendritic segments. 10 μm sampling area of each dendritic segment assayed was masked in SlideBook 6. Top panel shows manually generated mask using thresholding and the brush tool for “signal” of the dendritic segment. Middle panel shows the mask covering 100% of the pixels in the capture window. Bottom panel shows the mask created using the masks in the 2 panels on the left and Boolean math. This mask represents the “noise.” SNR was calculated as mean intensity of the signal divided by mean intensity of the noise. SNR for the segment in this example = 3.205. Segments with SNR > 2 were included in data analysis. Scalebar = 1 μm. (**E**) Assessment of NeuN labeling in 10x images were used to estimate region and determine laminar location of cell bodies of red fluorescent pyramidal cells. This example from region A2. Scalebar = 50 μm.

Free-floating sections corresponding to plates 55 and 59 in Franklin and Paxinos’s The Mouse Brain In Stereotaxic Coordinates^32^ were selected for immunohistochemistry. Plates 55 and 59 correspond to −2.92 mm and −3.4 mm from bregma, respectively. A1, A2, V1, V2 and TeA are each found at both of these stereotaxic coordinates. Free-floating sections were washed in 0.1 M PB to remove Tissue-Tek O.C.T. compound (Sakura Finetek Europe, Alphen aan den Rijn, Netherlands), then incubated for 30 m in 1% NaBH_4_ to reduce autofluorescence. After thorough rinsing, sections were blocked for 3 h in a solution of 1% normal goat serum, 3% Triton X-100, 1% bovine serum albumin, 0.1% lysine and 0.1% glycine. Sections were then incubated in the primary antibodies guinea pig anti-NeuN (Millipore ABN90 lot:2834791, 1:2000) and chicken anti-GFP (ThermoFisher A10262 lot: 1972783, 1:1000) for 24 h and 96 h respectively. Anti-NeuN was utilized to label neurons and Anti-GFP to amplify the eGFP signal. Pilot experiments demonstrated that amplifying the eGFP signal with a 568 secondary antibody rather than a 488 secondary antibody produced dendrites with superior signal-to-noise characteristics (Fig. 3A). Therefore, following primary antibody incubation, sections were washed and incubated in the secondary antibodies goat anti-guinea pig 405 (Abcam Ab175678 lot:1972783, 1:500) and goat anti-chicken, Alexa Fluor 568 (ThermoFisher A11041 lot:1963088, 1:500). After a 24 h incubation in secondary antibodies, sections were washed and mounted on TruBond 380 micro slide glass (Matsunami, Osaka, Japan) using ProLong Gold antifade mountant (Invitrogen, ThermoFisher Scientific, Waltham, MA).

### Sampling and confocal imaging

Images were captured using an Olympus BX51 WI upright microscope (Center Valley, PA) with an Olympus spinning disk confocal, Hamamatsu ORCA R2 CCD camera (Bridgewater, NJ), BioPrecision2 XYZ motorized stage with linear XYZ encoders (Ludl Electronic Products Ltd., Hawthorne, NY), Lumen 220 light source (Prior Scientific, Cambridge, United Kingdom), excitation and emission filterwheels (Ludl Electronic Products Ltd.) and a Sedat Quad 89000 filter set (Chroma Technology Corp., Bellows Falls, VT). 1.25 × 2-D images of each tissue section were acquired in SlideBook 6 software (Intelligent Imaging Innovations, Denver, CO) using 405 nm and 568 nm excitation. Franklin and Paxinos’s The Mouse Brain In Stereotaxic Coordinates^32^ was used to establish the region of interest (ROI) and estimate the regional location (A1, A2, V1, V2 or TeA) of the cell body of each pyramidal cell imaged (Fig. 3B). The region we define as A2 here includes both Franklin and Paxinos regions: secondary auditory cortex, dorsal region (2ary auditory cx, dorsal) and secondary auditory cortex, ventral region (2ary auditory cx, ventral). V1 refers to primary visual cortex, monocular region (primary visual cx, monocular) and primary visual cortex, binocular region (primary visual cx, binocular). Our definition of V2 includes all 3 subregions of secondary visual cortex (2ary visual cx, lat area, 2ary visual cx, mediolat and 2ary visual cx, mediomed). Collectively, the regions we assayed are primary and secondary auditory and visual cortices in ascending sensory pathways, with the exception of TeA, which is thought to be a multisensory region that processes complex auditory stimuli downstream from A1^44^. Fluorescent pyramidal cells transduced with AAV were identified at 1.25x magnification in *both hemispheres* and systematically numbered in the aforementioned 1.25 × 2-D image captures (Fig. 3B). Numbered cells were then randomly sampled and captured in 3-D image stacks using an Olympus PlanApo N 60 × /1.40 N.A. oil immersion super-corrected objective. Each capture site comprised of the cell body of one randomly selected (numbered) pyramidal cell, plus all basal dendrites visible within the capture window (Fig. 3C). Neutral density (ND) filter and exposure time for the 568 nm channel were optimized for one randomly selected minor basal dendritic segment at each site. Minor basal dendritic segment is defined here as any dendritic segment branching directly off of a major or primary basal dendrite. Total tissue thickness was estimated at each site by measuring anti-NeuN labeling in the z-dimension. 1024 × 1024 pixel 3-D image stacks were acquired through the entire thickness of the tissue (mean tissue thickness = 40.36 μm, standard deviation tissue thickness = 3.53 μm; 0.25 μm between each z-plane) in SlideBook 6 software.

### Image processing and analysis

SlideBook 6 and Stereo Investigator (MicroBrightField, Inc., Natick, MA) software were used for image processing and analysis. 1024 × 1024 image stacks were first transformed using a no-neighbors smoothing algorithm in SlideBook 6. All minor basal dendritic segments >10 μm away from the cell body and >3 μm from a dendrite branch point were identified in 1024 × 1024 image stacks (Fig. 3C) and cropped into individual image stacks containing one minor basal dendritic segment each. Minor basal dendritic segments were proximately located, with mean distance from soma 19.34 μm. Mean distance from soma was not significantly different across age or sex (data not shown). Signal-to-noise ratio (SNR) was calculated for each individual dendritic segment to compute fluorescent intensity of the dendritic segment (signal) relative to the background (noise) (Fig. 3D). The threshold for reliably distinguishing spines from fluorescent non-spine objects was set at SNR = 2. Segments that either failed to meet this SNR = 2 threshold or otherwise did not allow for reliable distinction between spines and non-spines were excluded. The length of each dendritic segment was measured in SlideBook 6 using the line tool. Individual dendritic segments were exported as TIFF series from SlideBook 6 and opened in Stereo Investigator for spine counting and categorization. Examination of anti-NeuN labeling was used to estimate laminar location, post-hoc^66,67,72^ (Fig. 3E).

Spine density for each neuron was calculated using the following equation: $Dendritic\,spine\,density\,(DSD)=$ $\frac{{\rm{total}}\,\#\,{\rm{dendritic}}\,{\rm{spines}}}{\Sigma \,dendrite\,lengths\,}$. In 1970 Peters and Kaiserman-Abramof introduced what is currently accepted as the traditional classification of morphological types: stubby, thin and mushroom dendritic spines^73^. We included these types in our analysis of dendritic protrusion morphology along with branched dendritic spines^76^, filopodia, and atypical dendritic spines^5^, a catch-all category which includes protrusions that do not conform to any of the aforementioned morphological types. Dendritic protrusions were manually counted and classified into one of eight types (short stubby, long stubby, short mushroom, long mushroom, thin, branched or atypical dendritic spine or filopodia) based on morphological characteristics described by other groups at length^5,73^ (Supplemental Fig. 2A). Short dendritic spines had maximal width greater than length, and long spines had maximal length greater than width. Mushroom spines had >0.4 μm head diameter with a clear distinction between spine head and neck. Stubby spines were dendritic protrusions with no significant distinction between head and neck. Thin spines had ≤0.3 μm head diameter and <2 μm total length. Branched spines had 2 spine heads attached to 1 spine neck. Atypical spines were <2 μm long and did not fall into any of the abovementioned types^5,73^. Filopodia were defined as >2 μm long dendritic protrusions with no distinction between spine head and neck^73^. Filopodia were not included in the total count of dendritic spines and thus not built into DSD for each neuron^74^. Long mushroom spines made up the highest proportion of dendritic protrusions counted in our study (66.03%). Long stubby spines accounted for 14.66%. The remaining dendritic protrusion types collectively accounted for less than 10% of the total spines counted, with filopodia accounting for 1.33%, thin spines 1.52%, and branched and atypical spines collectively making up <1% (Supplemental Fig. 2B).

### Statistics

Statistical tests were performed in SPSS software (IBM, Armonk, NY). The Shapiro-Wilk test was used to confirm normality. The Breusch-Pagan test was used to confirm that variances were equal regardless of age or sex. Tissue thickness, layer and region were built into ANCOVA models as covariates, and the effects of age, sex, region, layer and age by layer interaction, and significance were tested using a univariate general linear model. Total number of fluorescent cells in ROI, mean distance from soma, ND filter and 568 exposure time were identified as measures that did not significantly affect mean DSD and thus were not built into statistical models. Since layer was highly significant in the primary model, indicating layer strongly predicted mean DSD, this variable was included as a main effect in a secondary ANCOVA along with age and sex (with covariates: tissue thickness and region). Main effect of layer and age by layer, sex by layer and age by sex by layer interactions were Bonferroni corrected (p = 0.05) in the secondary model. Fitting the data to a mixed effects model with mouse as the random effect revealed that the within mouse correlation is not significant (p = 0.1125), ruling out the possibility that mean DSD of individual mice drove the group findings. Thus, DSD is reported throughout the paper at the level of individual neurons. In addition, mean DSD was calculated for each animal, and the descriptive statistics are provided in Fig. 1C. Group mean DSD was tested using an ANCOVA (covariate: mean tissue thickness) with main effects of age and sex and age by sex interaction. Levene’s Test was used to test the possibility that the variance among female mean DSD was significantly different than the variance among male mean DSD. The latter was used as a proxy to determine if estrous stage could underlie variability in female mouse mean DSD.

MANCOVA (α = 0.05) with Bonferroni correction was used to detect significant differences in mean densities among dendritic protrusion types. Main effects of age and sex, and age by sex interaction were tested. Tissue thickness, layer and region were built into a multivariate analysis of variance as covariates with eight dependent variables: short stubby, long stubby, thin, short mushroom, long mushroom, branched, atypical and filopodia densities and age and sex fixed factors.

## Supplementary information

Supplementary Information.
