# Author Correction: Sex differences in dendritic spine density and morphology in auditory and visual cortices in adolescence and adulthood

**DOI:** 10.1038/s41598-020-76802-y

**Published:** 2020-11-12

**Authors:** Emily M. Parker, Nathan L. Kindja, Claire E. J. Cheetham, Robert A. Sweet

**Affiliations:** 1 Center for Neuroscience, University of Pittsburgh, Pittsburgh, USA; 2 Translational Neuroscience Program, University of Pittsburgh, Pittsburgh, USA; 3 Department of Psychiatry, University of Pittsburgh, Pittsburgh, USA; 4 Department of Neurobiology, University of Pittsburgh, Pittsburgh, USA; 5 Center for the Neural Basis of Cognition, Pittsburgh, USA

Correction to: *Scientific Reports* 10.1038/s41598-020-65942-w, published online 10 June 2020

The original version of this Article contained errors.

In the Results section, under the subheading ‘Dendritic spine density is lower in females’,

“In contrast to this prediction, ANCOVA (α = 0.05) revealed a highly significant decrease in DSD of neurons from female, compared to male mice (F = 14.838, DF = 1, *p* < 0.001) in A1, A2, V1, V2 and TeA.”

now reads:

“In contrast to this prediction, ANCOVA (α = 0.05) revealed a highly significant decrease in DSD of neurons from female, compared to male mice (F = 14.838, DF = 3, *p* < 0.001) in A1, A2, V1, V2 and TeA.”

Additionally, in the legend of Figure 1,

“(**A**) DSD is significantly reduced in female, compared to male mice (F = 14.838, DF = 1, *p* < 0.001).”

now reads:

“(**A**) DSD is significantly reduced in female, compared to male mice (F = 14.838, DF = 3, *p* < 0.001).”

Furthermore, in the Discussion,

“Despite no apparent sex difference in DSD in schizophrenia in A1 in adult postmortem tissue, sex differences are well described in schizophrenia including mean age of onset and clinical presentation of sympoms^74,75^.”

now reads:

“Despite no apparent sex difference in DSD in schizophrenia in A1 in adult postmortem tissue, sex differences are well described in schizophrenia including mean age of onset and clinical presentation of symptoms^74,75^.”

Finally, References 76–81 were incorrectly listed as References 68–73, Reference 83 was incorrectly listed as Reference 74, Reference 71 was incorrectly listed as Reference 75, and Reference 82 was incorrectly listed as Reference 76.

In addition, References 68–70 and 72–75 were incorrectly omitted and are listed below:

68 Hackett, T. A. Anatomical organization of the auditory cortex. *Journal of the American Academy of Audiology* **19**, 774–779 (2008).
69 Homman-Ludiye, J. & Bourne, J. A. Mapping arealisation of the visual cortex of non-primate species: lessons for development and evolution. *Frontiers in Neural Circuits* **8**, https://doi.org/10.3389/fncir.2014.00079 (2014).
70 McKinney, B. C. *et al.* Density of small dendritic spines and microtubule-associated-protein-2 immunoreactivity in the primary auditory cortex of subjects with schizophrenia. *Neuropsychopharmacology* **44**, 1055–1061 (2019).
72 Sweet, R. A., Henteleff, R. A., Zhang, W., Sampson, A. R. & Lewis, D. A. Reduced dendritic spine density in auditory cortex of subjects with schizophrenia. *Neuropsychopharmacology* **34**, 374–398 (2009).
73 Shelton, M. A. *et al.* Loss of microtubule-associated protein 2 immunoreactivity linked to dendritic spine loss in schizophrenia. *Biological Psychiatry* **78**, 374–385 (2015).
74 Leung MD, D. A. & Chue MRC Psych, D. P. Sex differences in schizophrenia, a review of the literature. *Acta Psychiatrica Scandinavica* **101**, 3–38 (2000).
75 *Diagnostic and statistical manual of mental disorders: DSM-5®*. Arlington, VA: American Psychiatric Association (2013).

These errors have now been corrected in the HTML and PDF versions of the Article.

