# Supplementary material for: Sex differences in dendritic spine density and morphology in auditory and visual cortices in adolescence and adulthood
Source: Sci Rep. 2020 Jun 10;10:9442. doi: 10.1038/s41598-020-65942-w (PMC7287134; doi:10.1038/s41598-020-65942-w)
Supplement: Supplementary file 1 — Supplementary Information. [file 41598_2020_65942_MOESM1_ESM.pdf]

COVER PAGE – SUPPLEMENTARY INFORMATION

Title Sex differences in dendritic spine density and morphology in auditory and visual cortices in adolescence and adulthood

Authors Emily M. Parker [1,2,3], Nathan L. Kindja[2,3], Claire E.J. Cheetham[1,4,5], Robert A. Sweet[1,2,3]

Affiliations [1]Center for Neuroscience, University of Pittsburgh [2]Translational Neuroscience Program, University of Pittsburgh [3]Department of Psychiatry, University of Pittsburgh [4]Department of Neurobiology, University of Pittsburgh [5]Center for the Neural Basis of Cognition

Contact Information

Emily M. Parker	Biomedical Science Tower, W1601<br>3811 O'Hara Street<br>Pittsburgh PA, 15213	Emp92@pitt.edu
Nathan L. Kindja	Biomedical Science Tower, W1601<br>3811 O'Hara Street<br>Pittsburgh PA, 15213	Nlk29@pitt.edu
Claire E.J. Cheetham	Biomedical Science Tower, E1456<br>3811 O'Hara Street<br>Pittsburgh PA, 15213	Cheetham@pitt.edu
Robert A. Sweet*	Biomedical Science Tower, W1645<br>3811 O'Hara Street<br>Pittsburgh PA, 15213	Sweetra@upmc.edu

***Corresponding author**

Supplemental Figure 1

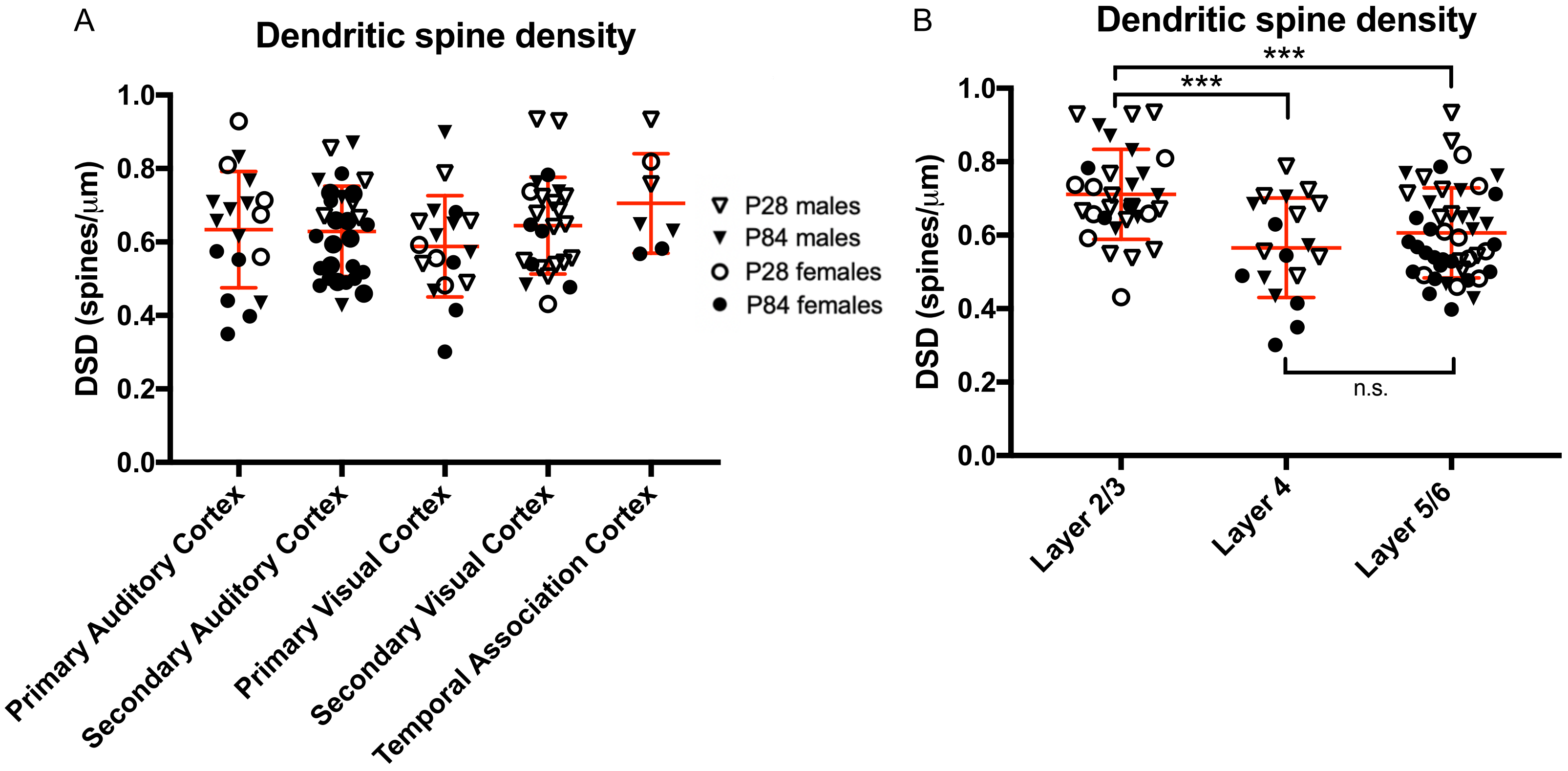

Supplemental Figure 2

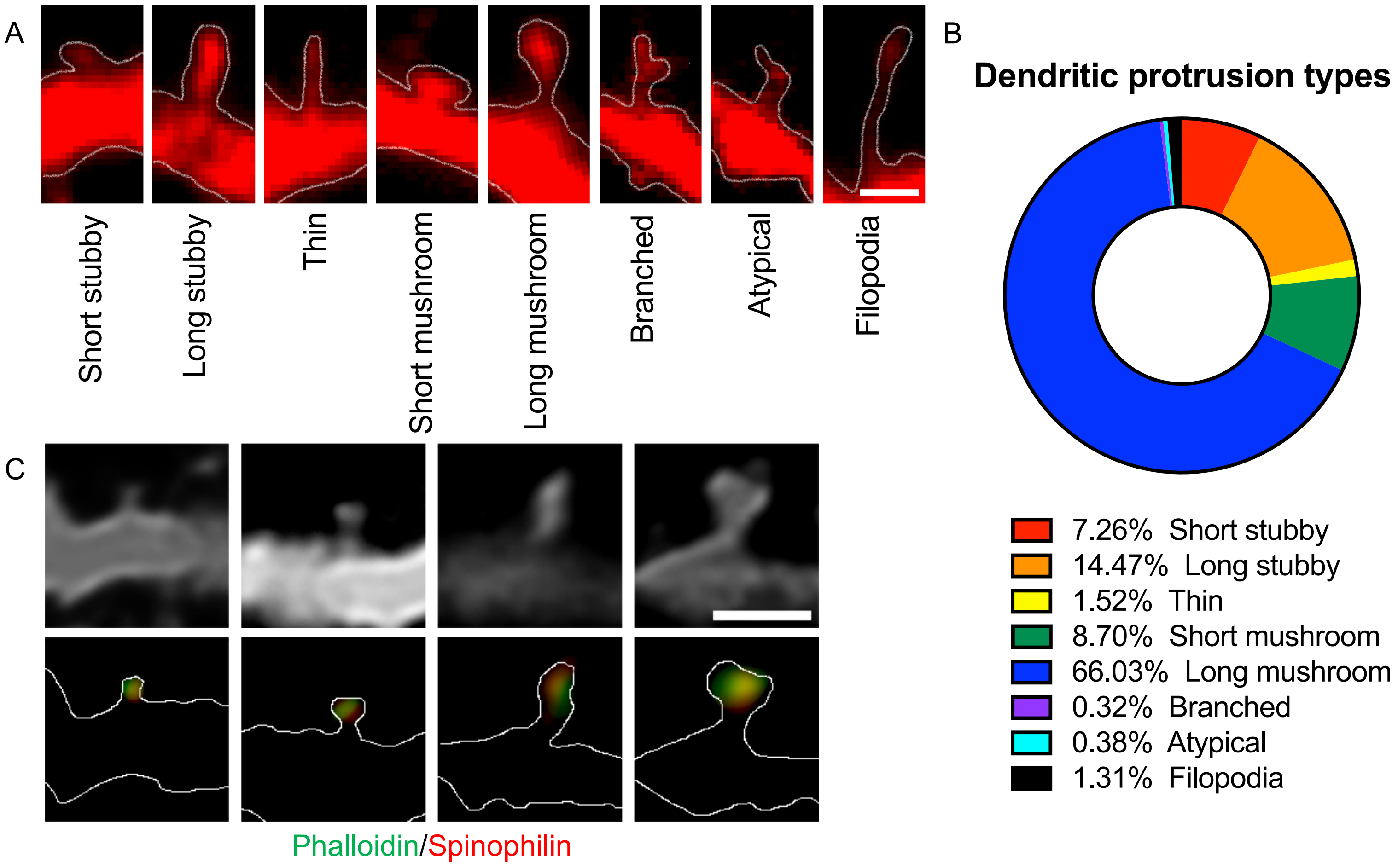

SUPPLEMENTAL FIGURE CAPTIONS

Supplemental Figure 1

Regional and laminar DSD. Mean DSD and SD represented by red lines. **A.** DSD is not significantly different across primary auditory, secondary auditory, primary visual, secondary visual and temporal association cortices ($F=1.829$, $DF=4$, $p=0.131$), with no significant region by sex nor region by age interactions. **B.** DSD is significantly increased in L2/3 compared to in L4 ($p<0.001$) and to in L5/6 ($p<0.001$). DSD in L4 and L5/6 are not significantly different ($p=0.707$).

Supplemental Figure 2

Dendritic protrusions. **A.** Depictions of each dendritic protrusion type. Scalebar = 1μm. **B.** All dendritic protrusions (n = 6241) were classified into 1 of the 8 morphological types shown in A. **C.** GFP filled dendrite (shown in white) from pyramidal cell in mouse cortex. Immunohistochemical strategy used in our previously published study [49], which utilized phalloidin and spinophilin co-localization to identify putative spine objects, identifies GFP-filled spines in the tissue used in the current study. Scalebar = 1μm.
